# Intraspecific Body Size Frequency Distributions of Insects

**DOI:** 10.1371/journal.pone.0016606

**Published:** 2011-03-30

**Authors:** E. Jeanne Gouws, Kevin J. Gaston, Steven L. Chown

**Affiliations:** 1 Department of Botany and Zoology, Centre for Invasion Biology, Stellenbosch University, Stellenbosch, South Africa; 2 Biodiversity and Macroecology Group, Department of Animal and Plant Sciences, University of Sheffield, Sheffield, United Kingdom; Dalhousie University, Canada

## Abstract

Although interspecific body size frequency distributions are well documented for many taxa, including the insects, intraspecific body size frequency distributions (IaBSFDs) are more poorly known, and their variation among mass-based and linear estimates of size has not been widely explored. Here we provide IaBSFDs for 16 species of insects based on both mass and linear estimates and large sample sizes (n≥100). In addition, we review the published IaBSFDs for insects, though doing so is complicated by their under-emphasis in the literature. The form of IaBSFDs can differ substantially between mass-based and linear measures. Nonetheless, in non-social insects they tend to be normally distributed (18 of 27 species) or in fewer instances positively skewed. Negatively skewed distributions are infrequently reported and log transformation readily removes the positive skew. Sexual size dimorphism does not generally cause bimodality in IaBSFDs. The available information on IaBSFDs in the social insects suggests that these distributions are usually positively skewed or bimodal (24 of 30 species). However, only *c*. 15% of ant genera are polymorphic, suggesting that normal distributions are probably more common, but less frequently investigated. Although only 57 species, representing seven of the 29 orders of insects, have been considered here, it appears that whilst IaBSFDs are usually normal, other distribution shapes can be found in several species, though most notably among the social insects. By contrast, the interspecific body size frequency distribution is typically right-skewed in insects and in most other taxa.

## Introduction

Body size is one of the most striking traits of all organisms. It is also one of the most significant. Strong relationships exist between body size and a variety of physiological and ecological features, including metabolic rate, production rate, survival probability, and the likelihood of dispersal [1], [2]. In turn, the size-dependencies of these characteristics influence body size over the short-term and on longer, evolutionary time-scales [3], [4]. They also affect the structure and dynamics of communities [5]. Much attention has thus been paid to understanding the physiological, ecological and evolutionary causes and consequences of body size variation. One of the most commonly used ways of investigating interactions between physiological and ecological determinants of body size and how these might result in evolutionary size change (or stasis) is by examination of the form of and influences on the size frequency distributions of organisms. Such approaches are common to life history theory [3], [6], [7], macroecology [8] and palaeobiology [9]. Indeed, intraspecific and interspecific body size frequency distributions have played important roles in the development of these fields, and particularly of macroecology, which regularly adopts univariate (i.e. frequency distribution-based), bivariate and then multivariate perspectives to understanding large-scale spatial and temporal variation in body size, range size and abundance [8].

From a macroecological perspective much is known about interspecific body size frequency distributions in vertebrates (summarized in [6], [8], [10]), and insects, where many studies have investigated their form, the mechanisms underlying them, and their broader consequences [5]. By contrast, despite the fact that intraspecific body size frequency distributions constitute a central component of macroecology [8], and are the outcome of the kinds of physiological and ecological interactions typically investigated to understand the causal basis for size variation generally [11], [12], they have not been widely documented for insects. Moreover, where this has been done, the focus of a given study has typically not been on the form of the body size frequency distribution, but rather the distribution is reported as one outcome of work that has had other goals [5]. The notable exception is work on social insects, and especially the ants, where frequency distributions of some, usually linear, measure of size are often provided to help understand the causes, consequences and evolution of the caste distribution function (or the relative sizes of ants in a given colony – for discussion see [3], [13]–[17]). Nonetheless, the recommendation is frequently made that for investigation of caste distribution functions and variation in polymorphism among species, static allometries (*sensu* [18]) be used in preference to size frequency distributions [13], [19]–[21].

Explicit investigation of the generality of the form of intraspecific body size frequency distributions is important from the life history and macroecological perspectives. Assessing the form of the distribution is a necessary first step in understanding the mechanisms that may generally underlie such distributions, across all taxa [6], [8], and provides specific information on what the ecological causes and consequences of the shapes and central tendencies of such distributions are likely to be (for insects see examples in [3], [22]–[25]). However, documentation of these distributions often neglects the fact that characterisation of their form can be confounded by several factors. In particular, though adult body mass may be a preferred metric for size, it may fluctuate considerably through time, especially in those insects which continue to grow in mass, but not linear dimensions, following eclosion, or in income-breeding species [26], [27]. Here, a linear measurement may be preferred, although this too may be confounded, such as by differences in shape or in allometry among the sexes. The characterization of size frequency distributions can also be confounded by statistical difficulties. These include those associated with selection of the number and range of the size classes used [28], and the effects of sampling season (insects show substantial seasonal variation in size [5], [29]) and geographic variation (given the often substantial size clines in widespread species – see review in [5], [30]). In this study, we therefore set out to investigate explicitly the form of the intraspecific body size frequency distribution (IaBSFD) in 16 insect species representing seven orders, paying particular attention to minimization of the above confounding effects. We examine these distributions using both linear dimensions and mass as estimates of body size, and do so for the distributions as a whole and for each sex separately. We then compare the outcome of these investigations with those undertaken previously (even where the IaBSFD was an incidental product of the study). We indicate what form of IaBSFD is typical of insects, the extent to which it might vary between mass and linear estimates of size, and how the IaBSFDs found for this group compare with those of other taxa.

## Methods

### Sampling and size estimates

Sampling was undertaken of the individuals of a given species (species were selected such that a sample size of ≥100 could be achieved), using the most effective technique for the group, and from a single location during the same day or week. All species were collected in the Western Cape Province of South Africa (Table 1) and returned to the laboratory within 2 h of collection. Animals were held in sampling jars humidified with moist filter paper and transported in insulated, cooled sampling containers. In the laboratory they were held at temperatures between 15°C and 20°C in their original sampling jars at low density (crowding can lead to cuticular damage and water loss [31]).

**Table 1:** The 16 insect species collected for this study indicating the linear measurement used to estimate size.

Order	Family	Species	Linear measure
Isoptera	Hodotermitidae	*Microhodotermes viator*	Body length
Orthoptera	Gryllidae	*Gryllus bimaculatus*	Maximum head width
Hemiptera	Lygaeidae	*Nysius* sp.	Body length
	Vellidae	*Rhagovelia maculata*	Body length
Lepidoptera	Satyridae	*Dira clytus*	Body length
Coleoptera	Apionidae	*Setapion provinciale*	Elytron length
		*Setapion quantillum*	Elytron length
	Chrysomelidae	Chrysomelid sp.	Body length
	Coccinellidae	*Henosepilachna vigintioctopunctata*	Elytron length
	Curculionidae	*Gonipterus scutellatus*	Elytron length
	Scarabaeidae	*Pachnoda sinuata*	Elytron length
Diptera	Tephritidae	*Ceratitis capitata*	Body length
Hymenoptera	Formicidae	Formicidae sp. alates	Head length
	Pteromalidae	*Trichilogaster acaciaelongifoliae*	Body length
	Pteromalidae	*Trichilogaster signiventris*	Body length
	Vespidae	*Polistes* sp.	Body length

All collections were made within a 10 km radius of the town of Stellenbosch (S33°55.92′ E18°51.80′), except for the termite species which was collected at Wolseley (S33°24.84′ E19°12.03′). Maximum body length excluding antennae, maximum elytron length, and head width or length were used as linear estimates of size in keeping with previous studies.

Body mass and a single body length measure were used to obtain size frequency distributions. The wet mass of the individuals of each species was determined using Mettler Toledo UMX2 or AX504 (Mettler-Toledo GmbH, Laboratory and Weighing Technologies, Greifensee, Switzerland) microbalances within 24 h of collection. Thereafter, the individuals were preserved (in alcohol or frozen) for future measurements. Body length or an appropriate surrogate variable (Table 1) were measured using a StereoLEICA MZ 7.5 (Leica Microsystems, Wetzlar, Germany) microscope, fitted with an ocular micrometer. Subsequently, the sex of each individual from each species was determined by dissection, to account for variance in body size between the sexes. This was not possible for four of the species (in *Polistes* sp. only females were collected, whereas in the *Nyssius* sp., *Setapion quantillum* and *Microhodotermes viator* the gender data were not collected). During the study, ten specimens of each species were measured repeatedly when 0%, 33%, 50%, 66% and 100% of all collected individuals of a species had been measured. This procedure was used to gauge the repeatability of the measurement process.

### Data analysis

Repeatability was determined using the intraclass correlation coefficient (*τ*), obtained from an analysis of variance (ANOVA, implemented in SAS version 9.1 (SAS Institute Inc., Cary, NC, USA)) and the equation for repeatability [32]. A *τ* value nearer to 1 implies that the measurement is accurate; while values nearer to 0 imply that the measurements are inaccurate, i.e. showing high variance for the same measurement.

To investigate intraspecific size frequency distributions of the species, both untransformed and log-transformed data were used for analysis. The log transformation was applied because it has been suggested that BSFDs should show a lognormal distribution [33]. Body size class (or bin size and number) is known to influence BSFDs [28]. Therefore, the number of bins for the BSFD of each species was chosen using Sturges' rule (*k = 1+log_2_n*). Although this may not always constitute the most appropriate approach, it has been found to be relatively effective for sample sizes that are smaller than 200.

Subsequently, deviation from normality of the mass and length distributions was established using the Shapiro-Wilks method. Furthermore, the significance of skew and kurtosis (sample statistic for skewness and kurtosis, *g_1_* and *g_2_*, respectively) was determined by t-test. Here a significant, positive *g_1_* value indicates that the distribution is right-skewed, and a significant negative *g_1_* value indicates a left skew. A negative *g_2_* indicates platykurtosis and a positive *g_2_* leptokurtosis. Owing to the possibility of an increase in the occurrence of Type I error, or false discovery rate, with repeated testing of data, the *P*-values obtained from the two-tailed t-tests were subjected to step-up FDR tests [34]. All statistical analyses were performed using the modelling program Enterprise Guide version 3.0, powered by SAS version 9.1 (SAS Institute Inc., Cary, NC, USA.). Significance was set at *P* = 0.05.

Within each species the extent of sexual size dimorphism was determined using generalised linear models (GENMOD procedure, SAS Institute Inc., Cary, NC, USA: GLZ, Type III models). Analyses of the frequency distributions were repeated for each sex separately where possible.

## Results

**Figure 1:**
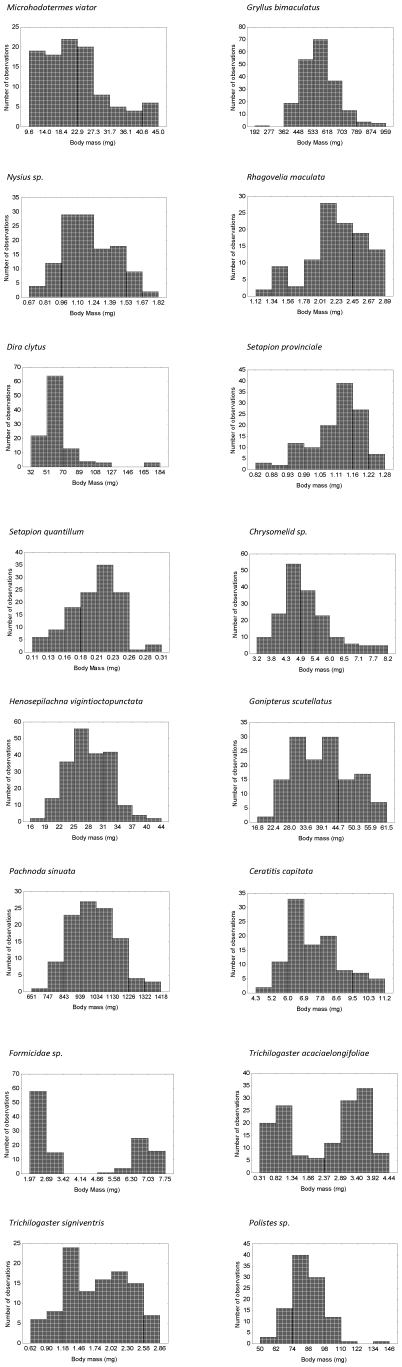
Body mass frequency distributions of the 16 species sampled for this study.

Significant repeatability estimates of greater than *τ* = 0.88 were obtained for all species and showed that the measurement process was precise. Considerable variation in the extent to which the mass and length distributions were normal or were skewed was found among the species. Thus, of the 16 species sampled, ten failed the test for normality in the case of the mass-based IaBSFDs. The untransformed IaBSFDs were significantly right-skewed in seven species, bimodal in two species, and one species had a significantly left-skewed distribution (Fig. 1, Table 2). In seven cases, log transformation of the data had no apparent effect on the distributions, and in four cases the right skew was removed after log transformation of the mass data (Table 3). Log transformation of the normally distributed data introduced significant negative skew in four cases (Table 3).

**Table 2:** Outcome of the assessment of deviation from normality (Shapiro-Wilks *W* statistic) and the degree of skewness (*g_1_*) and kurtosis (*g_2_*) for the untransformed body mass frequency distributions of all 16 insect species considered.

Species	n	*W*	*P*	*g_1_*	*g_2_*
*Microhodotermes viator*	102	0.923	<0.0001	0.872***	0.301^ns^
*Gryllus bimaculatus*	201	0.973	0.0006	0.537**	1.338**
*Nysius* sp.	120	0.989	0.477	0.227^ns^	−0.329^ns^
*Rhagovelia maculata*	108	0.953	0.0008	−0.694**	0.184^ns^
*Dira clytus*	109	0.663	<0.0001	3.174***	12.177***
*Setapion provinciale*	112	0.993	0.838	−0.102^ns^	0.257^ns^
*Setapion quantillum*	120	0.982	0.110	−0.265^ns^	0.181^ns^
Chrysomelid sp	175	0.929	<0.0001	1.024***	1.035^ns^
*Henosepilachna vigintioctopunctata*	207	0.988	0.073	0.333^ns^	0.438^ns^
*Gonipterus scutellatus*	138	0.979	0.032	0.229^ns^	−0.722^ns^
*Pachnoda sinuata*	108	0.994	0.920	0.165^ns^	−0.068^ns^
*Ceratitis capitata*	103	0.955	0.0015	0.559*	−0.318^ns^
Formicidae sp	120	0.753	<0.0001	0.508*	−1.657*
*Trichilogaster acaciaelongifoliae*	143	0.889	<0.0001	−0.260^ns^	−1.508*
*Trichilogaster signiventris*	107	0.976	0.051	−0.025^ns^	−0.912^ns^
*Polistes* sp.	103	0.951	0.0007	0.693**	3.211**

*P<0.05,

**P<0.01,

***P<0.001,

ns = not significant, after correction for the false discovery rate.

**Table 3:** Outcome of the assessment of deviation from normality (Shapiro-Wilks *W* statistic) and the degree of skewness (*g_1_*) and kurtosis (*g_2_*) for the log transformed body mass frequency distributions of all 16 insect species considered.

Species	n	*W*	*P*	*g_1_*	*g_2_*
*Microhodotermes viator*	102	0.981	0.152	0.042^ns^	−0.494^ns^
*Gryllus bimaculatus*	201	0.962	<0.0001	−0.607***	3.811***
*Nysius* sp.	120	0.989	0.420	−0.274^ns^	−0.058^ns^
*Rhagovelia maculata*	108	0.899	<0.0001	−1.226***	1.476**
*Dira clytus*	109	0.860	<0.0001	1.683***	4.534***
*Setapion provinciale*	112	0.952	0.0005	−1.00***	2.926***
*Setapion quantillum*	120	0.948	0.0001	−0.851***	0.755^ns^
Chrysomelid sp	175	0.975	0.003	0.484**	0.221^ns^
*Henosepilachna vigintioctopunctata*	207	0.991	0.263	−0.202^ns^	0.301^ns^
*Gonipterus scutellatus*	138	0.981	0.051	−0.291^ns^	−0.373^ns^
*Pachnoda sinuata*	108	0.992	0.805	−0.232^ns^	0.029^ns^
*Ceratitis capitata*	103	0.974	0.042	0.141^ns^	−0.378^ns^
Formicidae sp	120	0.779	<0.0001	0.412^ns^	−1.718*
*Trichilogaster acaciaelongifoliae*	143	0.859	<0.0001	−0.779***	−0.657^ns^
*Trichilogaster signiventris*	107	0.955	0.001	−0.649**	−0.059^ns^
*Polistes* sp.	103	0.961	0.0037	−0.279^ns^	1.949**

*P<0.05,

**P<0.01,

***P<0.001,

ns = not significant, after correction for the false discovery rate.

**Figure 2:**
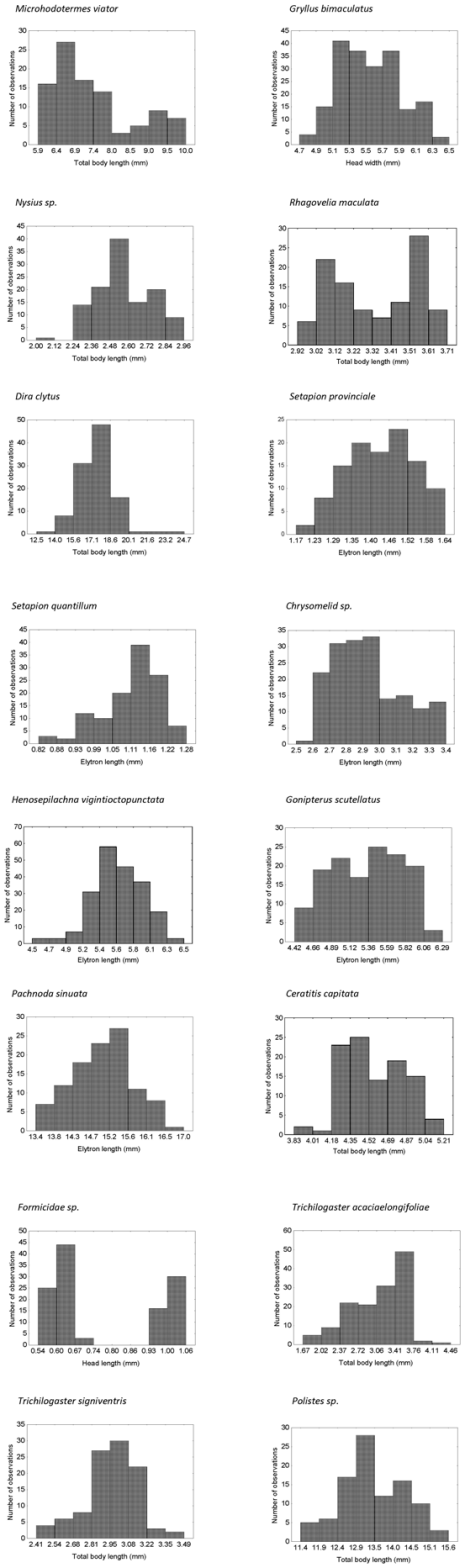
Body length frequency distributions of the 16 species sampled for this study. The length measurements were for different structures (see Table 1).

Of the IaBSFDs based on the linear measures, eleven showed no significant skew, three were significantly left-skewed, and two were significantly right-skewed. Most (12) distributions failed the test for normality and two were bimodal (Fig. 2, Table 4). The nature and extent of the skew for the linear measure was the same as for the mass-based IaBSFDs in seven of the species, but not in the others: this was reflected in a Pearson product-moment correlation coefficient of r = 0.687 (P = 0.03) between the *g_1_* values of the two sets of distributions. Log transformation of the length data had no obvious effect on the shape of the observed distributions in 13 species, whereas in the three others, log transformation served to increase the left-skew (Table 5). In most cases, kurtosis was not significant, and no further investigation of possible trends therein among the species was therefore undertaken.

**Table 4:** Outcome of the assessment of deviation from normality (Shapiro-Wilks *W* statistic) and the degree of skewness (*g_1_*) and kurtosis (*g_2_*) for the untransformed body size frequency distributions based on the linear measures made for all 16 insect species considered.

Species	n	*W*	*P*	*g_1_*	*g_2_*
*Microhodotermes viator*	98	0.894	<0.0001	0.826**	−0.453^ns^
*Gryllus bimaculatus*	199	0.971	0.0003	0.208^ns^	−0.567^ns^
*Nysius* sp.	120	0.980	0.071	0.094^ns^	−0.114^ns^
*Rhagovelia maculata*	108	0.903	<0.0001	−0.044^ns^	−1.506**
*Dira clytus*	107	0.937	<0.0001	0.728**	4.743***
*Setapion provinciale*	112	0.975	0.035	−0.194^ns^	−0.827^ns^
*Setapion quantillum*	120	0.952	0.0003	−0.819***	0.532^ns^
Chrysomelid sp	172	0.953	<0.0001	0.046^ns^	−0.367^ns^
*Henosepilachna vigintioctopunctata*	207	0.988	0.068	−0.226^ns^	0.529^ns^
*Gonipterus scutellatus*	138	0.965	0.0014	−0.107^ns^	−1.012^ns^
*Pachnoda sinuata*	107	0.987	0.417	0.044^ns^	−0.459^ns^
*Ceratitis capitata*	103	0.966	0.010	0.172^ns^	−0.341^ns^
Formicidae sp	119	0.766	<0.0001	0.412^ns^	−1.722**
*Trichilogaster acaciaelongifoliae*	140	0.940	<0.0001	−0.608**	−0.193^ns^
*Trichilogaster signiventris*	102	0.959	0.003	−0.530*	0.879^ns^
*Polistes* sp.	97	0.980	0.156	0.097^ns^	−0.315^ns^

*P<0.05,

**P<0.01,

***P<0.001,

ns = not significant, after correction for the false discovery rate.

**Table 5:** Outcome of the assessment of deviation from normality (Shapiro-Wilks *W* statistic) and the degree of skewness (*g_1_*) and kurtosis (*g_2_*) for the log transformed body size frequency distributions based on the linear measures made for all 16 insect species considered.

Species	n	*W*	*P*	*g_1_*	*g_2_*
*Microhodotermes viator*	98	0.921	<0.0001	0.633**	−0.656^ns^
*Gryllus bimaculatus*	199	0.973	0.0009	0.064^ns^	−0.590^ns^
*Nysius* sp.	120	0.980	0.065	−0.119^ns^	0.166^ns^
*Rhagovelia maculata*	108	0.903	<0.0001	−0.098^ns^	−1.475**
*Dira clytus*	107	0.953	0.0008	0.005^ns^	3.428***
*Setapion provinciale*	112	0.972	0.016	−0.328^ns^	−0.680^ns^
*Setapion quantillum*	120	0.928	<0.0001	−1.064***	1.206*
Chrysomelid sp	172	0.965	0.0002	0.444*	−0.480^ns^
*Henosepilachna vigintioctopunctata*	207	0.979	0.004	−0.464**	0.967*
*Gonipterus scutellatus*	138	0.962	0.0007	−0.231^ns^	−0.956^ns^
*Pachnoda sinuata*	107	0.987	0.0380	−0.070^ns^	−0.496^ns^
*Ceratitis capitata*	103	0.968	0.013	0.017^ns^	−0.211^ns^
Formicidae sp	119	0.781	<0.0001	0.363^ns^	−1.731**
*Trichilogaster acaciaelongifoliae*	140	0.908	<0.0001	−1.002***	0.601^ns^
*Trichilogaster signiventris*	102	0.944	0.0003	−0.779**	1.110^ns^
*Polistes* sp.	97	0.981	0.177	−0.072^ns^	−0.243^ns^

*P<0.05,

**P<0.01,

***P<0.001,

ns = not significant, after correction for the false discovery rate.

Ten of the 12 species for which data were available were sexually dimorphic on a mass basis, with females larger than males (Figs. S1 and S2 and Table S1). Similar, sex-related differences were found for the linear measurements, although in this case, dimorphism was not present in *S. provinciale* and *D. clytus*, and in *G. bimaculatus* males were larger, but here head width was used as the linear size estimate (Figure S2). Most of the IaBSFDs examined separately for the sexes did not show any significant deviations from normality in the case of body mass, although the distributions based on linear measurements tended not to be normal (Tables S2 and S3). Nonetheless, for both males and females, strong correlations were found between the skewness values for the linear and mass data (males r = 0.88, P<0.001; females r = 0.98, P<0.001). In the case of the body mass-based IaBSFD, seven of the 12 species showed no difference in the extent of skew among males and females, but overall, skewness was uncorrelated among the sexes (r = −0.18, P>0.57). Two species showed opposite skews. For example, the body mass distribution of the males of the butterfly species *D. clytus* was significantly left-skewed, whereas in the females it was significantly right-skewed (Table S2). A similar pattern was found for the linear estimates of size (here r among the sexes = −0.33, P>0.28). As was the case for the IaBSFDs generally, where a positive skew was present in the untransformed mass and/or length data, the skew was often removed after log transformation (Tables S2 and S3). Patterns in kurtosis were less consistent than for those in skew (Tables S2 and S3).

Intraspecific body size frequency distributions are available for a range of insects (Table S4), but they are often not reported explicitly. Rather, they usually form an under-emphasized part of an investigation into some other aspect of the biology of a given species, such as colony efficiency and polyethism in social insects [17], [23] or cryptic species [35], and therefore identifying their availability is not straightforward. Nonetheless, we found IaBSFDs for 57 species, including those examined here, and their joint consideration reveals several strong patterns. First, among social insects, positively skewed or bimodal distributions are commonly reported (80% of 30 species listed), with normal distributions characteristic of just three species. By contrast, among the remainder of the insects, normal distributions dominate, being characteristic of 67% of 27 species.

## Discussion

Although the current summary of data is unlikely to be comprehensive, given the wide variety of studies within which size-frequency distributions are reported and the common practise of not identifying their use in abstracts and keywords [e.g. 36]–[38], the scope of these appears nonetheless to be narrow relative to the diversity of the insects. Only seven of the *c*. 29 orders are represented, by 21 families and a tiny proportion (56 species) of the described fauna.

This said, several clear trends appear to emerge even from this relatively small sample of the group. Among the non-social species intraspecific body size distributions tend either to be normally distributed or slightly positively skewed, with few negatively skewed distributions and bimodality being relatively rare. The same pattern characterizes both mass-based and linear measures of body size, although the data collected for the 14 non-social species in this study suggest that a stronger tendency to a normal distribution exists in the linear measures. The extent to which the shapes of the distributions based on mass and linear measures will differ depends on whether the relationship between the two kinds of measures is isometric or allometric. At least among linear measures the relationship is frequently allometric, resulting in substantial variation in the extent of polymorphism among structures, including complex variation associated with sigmoidal or discontinuous static allometries, such as found in beetle horn polymorphisms [18], [39]. The relationships within species among the linear traits typically used to characterize body size and body mass are frequently not isometric [40], suggesting that measures of the form of distributions will differ among the traits. This was the case here, for skewness for example, even though the skewness measures were significantly correlated across the two measures (r being below 0.7). In consequence, the choice of a feature to characterize the size frequency distribution of a particular population needs to take into consideration the form of static allometries likely to be found in the group, the particular goals of the study, as well as the practicability of data acquisition. The latter might be especially important, for example, when museum specimens are being used to assess long-term changes in the shape or body size of the population of a particular species [41]. Perhaps the most appropriate approach would be to assess in a pilot trial a variety of measures to understand the nature of the static allometries in the species (or population), if these are not known already from similar work on related groups, and also to assess the relationship among the preferred linear measure and body mass in a subsample.

Although the majority of the non-social species examined here are characterized by sexual size dimorphism (with males smaller than females as is found in insects generally [12]), this did not typically result in dimorphic size frequency distributions except in one instance where males and females show limited overlap in mass (Fig. 1). Rather, the size overlap among the sexes simply contributes to the overall form of the distribution. Nonetheless, when the sexes are distinguished, the distributions tended to be normal, with much stronger relationships between the mass-based and linear estimates of skew than found in the case of the entire distributions. By contrast, very little relationship was found between skewness among the sexes irrespective of the measurement approach. Thus, although sexing individuals provides additional information on the way in which a population's size frequency distribution is constructed, it is not especially necessary for this information to be available in macroecological studies that seek to characterize the intraspecific body mass frequency distribution on a mass or linear basis. However, as with the previous discussion of linear measures, where complex static allometries exist such that characters may be exaggerated in one sex relative to the other, or may show much more variation in one sex relative to the other [39], these influences need to be excluded if the aim of the study is to examine body size as part of a macroecological investigation. Alternatively, if size optimization is at issue, the relationships between selection on size overall and on size of particular morphological features need to be taken into account [3], [39].

The identification and characterization of static allometries forms the basis for the recognition of substantial polymorphism, associated with castes and not with the sexes, within the social insects and especially the ants. In an early study, Wilson [13] recognized four forms of allometry: monophasic – a single slope for the regression, resulting in a positive skew or weak bimodality; diphasic – the regression line has two slope values with a break between them, also resulting in strongly skewed distributions and bimodality; triphasic – three slopes with two breaks, resulting in strong bimodality; and finally complete dimorphism. This approach has been criticized [42], but is still widely adopted and recommended [20], [21]. From the summary data presented here on the social insects it would appear that skewed or bimodal distributions are most characteristic of the group. Thus, of the 20 ant species listed in Table S4, 80% had positively skewed or bimodal distributions, and 75% of the eight species of bees showed positively skewed distributions. Only a single social wasp and one termite species were represented, but their distributions were likewise skewed. Moreover, much of the literature on ants seems to be concerned with how such a positive skew or bimodal distribution might develop as colonies age, or how the caste polymorphism might be maintained [22], [43]. Nonetheless, Wilson [13] suggested that most ant species are monomorphic, illustrating this with *Formica exsectoides*. Later, Oster and Wilson [3] argued that perhaps 15% of ant genera show size polymorphism, with the remainder having a monomorphic frequency distribution (the reproductives which tend to be larger in social insects are excluded from the assessment). Oster and Wilson [3] also provided a model of the costs of worker production vs. the distribution of resources in the environment to show that the evolution of polymorphism was likely to be infrequent. Much as their arguments have subsequently been debated, and the causes and consequences of polymorphism comprehensively assessed [21], [22], it seems that the situation of a relative paucity of skewed or bimodal distributions remains the norm for ants. It is not clear what the usual situation is for bees, but nest site selection has an effect on body size variation [44] as might recruitment system [45]. Nonetheless, bimodal distributions are not common.

If the majority of ants and other social insects have monomorphic, largely normal frequency distributions, based on linear measures, and presuming that this translates to body mass, then the data available suggest that among the insects normally distributed IaBSFDs are most common. Although assumed by much of life history theory [46], [47], it is also clear that not all species have such distributions, and that even within relatively monomorphic species IaBSFDs can change markedly over time [48]. IaBSFDs are also quite variable amongst other groups of organisms [49]–[52] although often appearing approximately normal or at least symmetrical. Several models have shown how the size dependencies of production and mortality may lead from relatively normal IaBSFDs to right-skewed interspecific frequency distributions [6], [46]. Indeed, the right-skewed interspecific frequency distribution is characteristic of most taxa at a broad range of scales [8], [11], only becoming less skewed, and sometimes more platykurtic, in more narrowly defined taxonomic groups or at smaller spatial scales (e.g. habitat rather than continent) [53]–[55]. The interspecific body size frequency distribution differs substantially from the intraspecific body size frequency distribution in this respect. The distributions also differ by virtue of there being no optimum body size for a given higher taxon, whereas a range of life-history models demonstrate clearly how, within a given population, an optimum body size is likely to evolve [3], [4].

## Supporting Information

Figure S1 **Body mass (mg) frequency distributions of males and females separately for 12 of the insect species considered.** The distributions for the females are presented on the left and the male distributions are on the right. The distributions are as follows; (**a**) *Gryllus bimaculatus* females and (**b**) males, (**c**) *Dira clytus* females and (**d**) males, (**e**) the ant species females and (**f**) males, (**g**) *Rhagovelia imaculata* females and (**h**) males, (**i**) *Setapion provinciale* females and (**j**) males, (**k**) the chrysomelid species females and (**l**) males, (**m**) *Ceratitis capitata* females and (**n**) males, (**o**) *Henosepilachna vigintioctopunctata* females and (**p**) males, (**q**) *Trichilogaster acaciaelongifoliae* females and (**r**) males, (**s**) *Trichilogaster signiventris* females and (**t**) males, (**u**) *Pachnoda sinuata* females and (**v**) males, and (**w**) *Gonipterus scutelatus* females and (**x**) males.
(DOC)

Figure S2 **Body length (mm) frequency distributions of males and females separately for 12 of the insect species considered.** The distributions for the females are presented on the left and the male distributions are on the right. The distributions are as follows; (**a**) *Gryllus bimaculatus* females and (**b**) males, (**c**) *Dira clytus* females and (**d**) males, (**e**) the ant species females and (**f**) males, (**g**) *Rhagovelia imaculata* females and (**h**) males, (**i**) *Setapion provinciale* females and (**j**) males, (**k**) the chrysomelid species females and (**l**) males, (**m**) *Ceratitis capitata* females and (**n**) males, (**o**) *Henosepilachna vigintioctopunctata* females and (**p**) males, (**q**) *Trichilogaster acaciaelongifoliae* females and (**r**) males, (**s**) *Trichilogaster signiventris* females and (**t**) males, (**u**) *Pachnoda sinuata* females and (**v**) males, and (**w**) *Gonipterus scutelatus* females and (**x**) males.
(DOC)

Table S1 **Mean (± s.e.) mass (mg) for each sex, the chi-squared and p values from a generalized linear model (normal distribution, identity link function) investigating sex-related size differences, and sample sizes (in parentheses) in each case.** Where the sex was not determined the data are shown in the centre of the two columns.
(DOC)

Table S2 **Outcome of the tests for the deviation from normality (Shapiro-Wilks** ***W*** **statistic) and the degree of skewness (** ***g_1_*** **) and kurtosis (** ***g_2_*** **) for the (a) untransformed body mass (mg) and (b) log transformed body mass frequency distributions of the males and females.** * P<0.05, ** P<0.01, *** P<0.001, ns = not significant, after correction for the false discovery rate.
(DOC)

Table S3 **Outcome of the tests for the deviation from normality (Shapiro-Wilks** ***W*** **statistic) and the degree of skewness (** ***g_1_*** **) for the (a) untransformed linear (mm) and (b) log transformed linear frequency distributions of the males and females.** * P<0.05, ** P<0.01, *** P<0.001, ns = not significant, after correction for the false discovery rate.
(DOC)

Table S4 **Intraspecific body size frequency distribution (IaBSFD) data for insects, indicating whether one or more figures of the data are provided, the outcome of tests for skewness, or normality if former not available, and whether additional data on variation associated with age, sex, time or space are provided.**
(DOC)
