# Supplementary material for: Intraspecific Body Size Frequency Distributions of Insects
Source: PLoS One. 2011 Mar 30;6(3):e16606. doi: 10.1371/journal.pone.0016606 (PMC3068144; doi:10.1371/journal.pone.0016606)
Supplement: Figure S2 — Body length (mm) frequency distributions of males and females separately for 12 of the insect species considered. The distributions for the females are presented on the left and the male distributions are on the right. The distributions are as follows; (a) Gryllus bimaculatus females and (b) males, (c) Dira clytus females and (d) males, (e) the ant species females and (f) males, (g) Rhagovelia imaculata females and (h) males, (i) Setapion provinciale females and (j) males, (k) the chrysomelid species females and (l) males, (m) Ceratitis capitata females and (n) males, (o) Henosepilachna vigintioctopunctata females and (p) males, (q) Trichilogaster acaciaelongifoliae females and (r) males, (s) Trichilogaster signiventris females and (t) males, (u) Pachnoda sinuata females and (v) males, and (w) Gonipterus scutelatus females and (x) males. (DOC) [file pone.0016606.s002.doc]

**2a**

**2c**

**2e**

**2b**

**2d**

**2f**

**2g**

**2i**

**2k**

**2h**

**2j**

**2l**

**2m**

**2o**

**2q**

**2n**

**2p**

**2r**

**2s**

**2u**

**2w**

**Supporting Information Figure S2.** Body length (mm) frequency distributions of males and females separately for 12 of the insect species considered. The distributions for the females are presented on the left and the male distributions are on the right. The distributions are as follows; **(a)** *Gryllus bimaculatus* females and **(b)** males, **(c)** *Dira clytus* females and **(d)** males, **(e)** the ant species females and **(f)** males, **(g)** *Rhagovelia imaculata* females and **(h)** males, **(i)** *Setapion provinciale* females and **(j)** males, **(k)** the chrysomelid species females and **(l)** males, (**m)** *Ceratitis capitata* females and **(n)** males, **(o)** *Henosepilachna vigintioctopunctata* females and **(p)** males, **(q)** *Trichilogaster acaciaelongifoliae* females and **(r)** males, **(s)** *Trichilogaster signiventris* females and **(t)** males, **(u)** *Pachnoda sinuata* females and **(v)** males, and **(w)** *Gonipterus scutelatus* females and **(x)** males.

**2t**

**2v**

**2x**
