# Supplementary material for: Intraspecific Body Size Frequency Distributions of Insects
Source: PLoS One. 2011 Mar 30;6(3):e16606. doi: 10.1371/journal.pone.0016606 (PMC3068144; doi:10.1371/journal.pone.0016606)
Supplement: Table S1 — Mean (± s.e.) mass (mg) for each sex, the chi-squared and p values from a generalized linear model (normal distribution, identity link function) investigating sex-related size differences, and sample sizes (in parentheses) in each case. Where the sex was not determined the data are shown in the centre of the two columns. (DOC) [file pone.0016606.s003.doc]

**Supporting Information Table S1.** Mean (± s.e.) mass (mg) for each sex, the chi-squared and p values from a generalized linear model (normal distribution, identity link function) investigating sex-related size differences, and sample sizes (in parentheses) in each case. Where the sex was not determined the data are shown in the centre of the two columns.

| **Species** | **Female** | **Male** | **χ^2^** | **P** |
| --- | --- | --- | --- | --- |
| *Microhodotermes viator* | 22.39 ± 0.86 (102) | | - | - |
| *Gryllus bimaculatus* | 580.51 ± 10.95 (106) | 561.78 ± 10.89 (95) | 1.47 | 0.225 |
| *Nysius* sp. | 1.20 ± 0.02 (120) | | - | - |
| *Rhagovelia maculata* | 2.41 ± 0.05 (56) | 1.99 ± 0.04 (52) | 35.9 | 0.00001 |
| *Dira clytus* | 78.43 ± 3.95 (54) | 53.44 ± 0.74 (55) | 34.0 | 0.00001 |
| *Setapion provinciale* | 0.60 ± 0.01 (59) | 0.60 ± 0.02 (53) | 0.06 | 0.81 |
| *Setapion quantillum* | 0.206 ± 0.003 (120) | | - | - |
| Chrysomelid sp | 5.77 ± 0.15 (63) | 4.69 ± 0.05 (109) | 56.5 | 0.00001 |
| *Henosepilachna vigintioctopunctata* | 30.54 ± 0.41 (106) | 26.37 ± 0.39 (100) | 48.0 | 0.00001 |
| *Gonipterus scutellatus* | 46.59 ± 0.87 (69) | 31.75 ± 0.66 (69) | 118.3 | 0.00001 |
| *Pachnoda sinuata* | 1059 ± 19 (61) | 962 ± 17 (46) | 13.0 | 0.0003 |
| *Ceratitis capitata* | 8.50 ± 0.19 (52) | 6.41 ± 0.08 (51) | 74.9 | 0.00001 |
| Formicidae sp | 6.80 ± 0.07 (46) | 2.46 ± 0.03 (73) | 420.5 | 0.00001 |
| *Trichilogaster acaciaelongifoliae* | 3.31 ± 0.06 (89) | 0.96 ± 0.04 (54) | 274.7 | 0.00001 |
| *Trichilogaster signiventris* | 2.17 ± 0.05 (59) | 1.31 ± 0.04 (48) | 107.7 | 0.00001 |
| *Polistes* sp. | 84.20 ± 1.37 (103) | No males | - | - |
