# Supplementary material for: Intraspecific Body Size Frequency Distributions of Insects
Source: PLoS One. 2011 Mar 30;6(3):e16606. doi: 10.1371/journal.pone.0016606 (PMC3068144; doi:10.1371/journal.pone.0016606)
Supplement: Table S2 — Outcome of the tests for the deviation from normality (Shapiro-Wilks W statistic) and the degree of skewness ( g1 ) and kurtosis ( g2 ) for the (a) untransformed body mass (mg) and (b) log transformed body mass frequency distributions of the males and females. * P<0.05, ** P<0.01, *** P<0.001, ns = not significant, after correction for the false discovery rate. (DOC) [file pone.0016606.s004.doc]

**Supporting Information Table S2.** Outcome of the tests for the deviation from normality (Shapiro-Wilks *W* statistic) and the degree of skewness (*g_1_*) and kurtosis (*g_2_*) for the **(a)** untransformed body mass (mg) and **(b)** log transformed body mass frequency distributions of the males and females. * P < 0.05, ** P < 0.01, *** P < 0.001, ns = not significant, after correction for the false discovery rate.

(a)

| **Species** | **W** | **P** | ***g_1_*** | ***g_2_*** | **W** | **P** | ***g_1_*** | ***g_2_*** |
| --- | --- | --- | --- | --- | --- | --- | --- | --- |
|  | **Males** |  |  |  | **Females** |  |  |  |
| *Gryllus bimaculatus* | 0.983 | 0.254 | -0.086 ^ns^ | 1.099 ^ns^ | 0.943 | 0.0002 | 0.981*** | 1.264* |
| *Rhagovelia maculata* | 0.832 | <0.0001 | -1.311*** | 0.786 ^ns^ | 0.845 | <0.0001 | -1.630*** | 2.746** |
| *Dira clytus* | 0.937 | 0.006 | -1.069** | 2.681* | 0.682 | <0.0001 | 2.523*** | 6.614*** |
| *Setapion provinciale* | 0.971 | 0.224 | -0.420 ^ns^ | 1.114 ^ns^ | 0.988 | 0.803 | 0.184 ^ns^ | -0.439 ^ns^ |
| Chrysomelid sp | 0.991 | 0.680 | -0.031 ^ns^ | -0.004 ^ns^ | 0.975 | 0.238 | 0.192 ^ns^ | -0.693 ^ns^ |
| *Henosepilachna vigintioctopunctata* | 0.983 | 0.206 | 0.213 ^ns^ | -0.306 ^ns^ | 0.975 | 0.044 | 0.500* | 1.000 ^ns^ |
| *Gonipterus scutellatus* | 0.983 | 0.447 | -0.106 ^ns^ | -0.321 ^ns^ | 0.982 | 0.399 | -0.111 ^ns^ | -0.341 ^ns^ |
| *Pachnoda sinuata* | 0.954 | 0.068 | -0.143 ^ns^ | -0.948 ^ns^ | 0.993 | 0.980 | -0.078 ^ns^ | 0.013 ^ns^ |
| *Ceratitis capitata* | 0.930 | 0.005 | 1.209 *** | 3.471** | 0.946 | 0.020 | -0.589 ^ns^ | 1.749** |
| Formicidae sp | 0.977 | 0.200 | 0.436 ^ns^ |  | 0.979 | 0.557 | -0.314 ^ns^ | 0.297 ^ns^ |
| *Trichilogaster acaciaelongifoliae* | 0.984 | 0.676 | 0.121 ^ns^ | -0.510 ^ns^ | 0.973 | 0.061 | -0.537* | 0.286 ^ns^ |
| *Trichilogaster signiventris* | 0.977 | 0.468 | -0.343 ^ns^ | -0.176 ^ns^ | 0.981 | 0.479 | -0.366 ^ns^ | -0.053 ^ns^ |

(b)

| **Species** | **W** | **P** | ***g_1_*** | ***g_2_*** | **W** | **P** | ***g_1_*** | ***g_2_*** |
| --- | --- | --- | --- | --- | --- | --- | --- | --- |
|  | **Males** |  |  |  | **Females** |  |  |  |
| *Gryllus bimaculatus* | 0.916 | <0.0001 | -1.404*** | 1.099 ^ns^ | 0.984 | 0.214 | 0.439 ^ns^ | 0.103 ^ns^ |
| *Rhagovelia maculata* | 0.788 | <0.0001 | -1.578*** | 1.731** | 0.761 | <0.0001 | -2.214*** | 5.557*** |
| *Dira clytus* | 0.880 | <0.0001 | -1.733*** | 5.888*** | 0.833 | <0.0001 | 1.595*** | 3.111** |
| *Setapion provinciale* | 0.892 | 0.0002 | -1.583 *** | 5.232*** | 0.986 | 0.741 | -0.261^ns^ | -0.299 ^ns^ |
| Chrysomelid sp | 0.983 | 0.167 | -0.395 ^ns^ | 0.215 ^ns^ | 0.978 | 0.306 | -0.219^ns^ | -0.543 ^ns^ |
| *Henosepilachna vigintioctopunctata* | 0.983 | 0.235 | -0.202 ^ns^ | 0.129 ^ns^ | 0.987 | 0.364 | -0.045 ^ns^ | 0.737 ^ns^ |
| *Gonipterus scutellatus* | 0.965 | 0.049 | -0.644* | 0.832 ^ns^ | 0.966 | 0.057 | -0.514 ^ns^ | 0.056^ns^ |
| *Pachnoda sinuata* | 0.947 | 0.035 | -0.319 ^ns^ | -0.924 ^ns^ | 0.978 | 0.334 | -0.543 ^ns^ | 0.757^ns^ |
| *Ceratitis capitata* | 0.960 | 0.082 | 0.797* | 2.103** | 0.871 | <0.0001 | -1.561*** | 4.870*** |
| Formicidae sp | 0.987 | 0.643 | 0.171 ^ns^ | -0.274 ^ns^ | 0.968 | 0.228 | -0.567 ^ns^ | 0.904 ^ns^ |
| *Trichilogaster acaciaelongifoliae* | 0.951 | 0.027 | -0.803* | 0.588 ^ns^ | 0.927 | <0.0001 | -1.085*** | 1.409** |
| *Trichilogaster signiventris* | 0.934 | 0.010 | -0.924* | 0.560 ^ns^ | 0.949 | 0.015 | -0.833* | -0.053 ^ns^ |
