# Supplementary material for: Intraspecific Body Size Frequency Distributions of Insects
Source: PLoS One. 2011 Mar 30;6(3):e16606. doi: 10.1371/journal.pone.0016606 (PMC3068144; doi:10.1371/journal.pone.0016606)
Supplement: Table S3 — Outcome of the tests for the deviation from normality (Shapiro-Wilks W statistic) and the degree of skewness ( g1 ) for the (a) untransformed linear (mm) and (b) log transformed linear frequency distributions of the males and females. * P<0.05, ** P<0.01, *** P<0.001, ns = not significant, after correction for the false discovery rate. (DOC) [file pone.0016606.s005.doc]

**Supporting Information Table S3.** Outcome of the tests for the deviation from normality (Shapiro-Wilks *W* statistic) and the degree of skewness (*g_1_*) for the **(a)** untransformed linear (mm) and **(b)** log transformed linear frequency distributions of the males and females. * P < 0.05, ** P < 0.01, *** P < 0.001, ns = not significant, after correction for the false discovery rate.

(a)

| **Species** | **W** | **P** | ***g_1_*** | ***g_2_*** | **W** | **P** | ***g_1_*** | ***g_2_*** |
| --- | --- | --- | --- | --- | --- | --- | --- | --- |
|  | **Males** |  |  |  | **Females** |  |  |  |
| *Gryllus bimaculatus* | 0.976 | 0.083 | -0.126 ^ns^ | -0.515 ^ns^ | 0.955 | 0.001 | 0.380 ^ns^ | -0.295 ^ns^ |
| *Rhagovelia maculata* | 0.947 | 0.021 | -0.327 ^ns^ | -0.122 ^ns^ | 0.910 | 0.0005 | -1.058** | 2.299** |
| *Dira clytus* | 0.968 | 0.163 | -0.126 ^ns^ | 1.208 ^ns^ | 0.941 | 0.011 | 0.609 ^ns^ | 3.464*** |
| *Setapion provinciale* | 0.950 | 0.027 | -0.219 ^ns^ | -1.120 ^ns^ | 0.986 | 0.729 | -0.148 ^ns^ | -0.640 ^ns^ |
| Chrysomelid sp | 0.975 | 0.975 | -0.218 ^ns^ | -0.505 ^ns^ | 0.950 | 0.012 | -0.612 ^ns^ | -0.184 ^n^s |
| *Henosepilachna vigintioctopunctata* | 0.934 | <0.0001 | -0.676* | 2.719** | 0.969 | 0.013 | -0.509** | 1.624** |
| *Gonipterus scutellatus* | 0.965 | 0.050 | 0.111 ^ns^ | -0.765 ^ns^ | 0.962 | 0.036 | -0.170 ^ns^ | -0.614 ^ns^ |
| *Pachnoda sinuata* | 0.964 | 0.165 | 0.291 ^ns^ | -0.726 ^ns^ | 0.978 | 0.334 | -0.340 ^ns^ | 0.606 ^ns^ |
| *Ceratitis capitata* | 0.929 | 0.004 | -0.441 ^ns^ | 0.902 ^ns^ | 0.925 | 0.003 | -1.014* | 2.331** |
| Formicidae sp | 0.960 | 0.021 | 0.059 ^ns^ | 0.231 ^ns^ | 0.947 | 0.037 | 0.072 ^ns^ | -0.707 ^ns^ |
| *Trichilogaster acaciaelongifoliae* | 0.920 | 0.001 | 1.135** | 4.891*** | 0.947 | 0.001 | -0.626* | -0.365 ^ns^ |
| *Trichilogaster signiventris* | 0.937 | 0.016 | -0.729 ^ns^ | -0.318 ^ns^ | 0.949 | 0.018 | -0.358 ^ns^ | -0.770 ^ns^ |

(b)

| **Species** | **W** | **P** | ***g_1_*** |  | **W** | **P** | ***g_1_*** |  |
| --- | --- | --- | --- | --- | --- | --- | --- | --- |
|  | **Males** |  |  |  | **Females** |  |  |  |
| *Gryllus bimaculatus* | 0.973 | 0.049 | -0.284 ^ns^ | -0.382 ^ns^ | 0.960 | 0.003 | 0.243 ^ns^ | -0.403 ^ns^ |
| *Rhagovelia maculata* | 0.944 | 0.016 | -0.397 ^ns^ | -0.0638 ^ns^ | 0.900 | 0.0002 | -1.190*** | 2.851** |
| *Dira clytus* | 0.963 | 0.091 | -0.417 ^ns^ | 1.414 ^ns^ | 0.953 | 0.038 | -0.107 ^ns^ | 2.636** |
| *Setapion provinciale* | 0.946 | 0.019 | -0.312 ^ns^ | -1.057 ^ns^ | 0.982 | 0.517 | -0.307 ^ns^ | -0.497 ^ns^ |
| Chrysomelid sp | 0.972 | 0.023 | -0.310 ^ns^ | -0.384 ^ns^ | 0.938 | 0.004 | -0.758* | 0.090 ^ns^ |
| *Henosepilachna vigintioctopunctata* | 0.918 | <0.0001 | -0.983*** | 3.334*** | 0.956 | 0.001 | -0.756** | 2.245** |
| *Gonipterus scutellatus* | 0.967 | 0.062 | 0.007 ^ns^ | -0.736 ^ns^ | 0.960 | 0.025 | -0.257 ^ns^ | -0.597 ns |
| *Pachnoda sinuata* | 0.967 | 0.210 | 0.220 ^ns^ | -0.771 ^ns^ | 0.971 | 0.157 | -0.505 ^ns^ | 0.842 ns |
| *Ceratitis capitata* | 0.924 | 0.003 | -0.574 ^ns^ | 1.276 ^ns^ | 0.903 | 0.0005 | -1.291*** | 3.424*** |
| Formicidae sp | 0.960 | 0.021 | -0.111 ^ns^ | 0.153 ^ns^ | 0.948 | 0.039 | 0.015 ^ns^ | -0.687 ^ns^ |
| *Trichilogaster acaciaelongifoliae* | 0.962 | 0.084 | 0.095 ^ns^ | 1.920** | 0.934 | 0.0003 | -0.748** | -0.198 ^ns^ |
| *Trichilogaster signiventris* | 0.921 | 0.005 | -0.894* | -0.392 ^ns^ | 0.940 | 0.007 | -0.634 ^ns^ | -0.680 ^ns^ |
