# Supplementary material for: Intraspecific Body Size Frequency Distributions of Insects
Source: PLoS One. 2011 Mar 30;6(3):e16606. doi: 10.1371/journal.pone.0016606 (PMC3068144; doi:10.1371/journal.pone.0016606)
Supplement: Table S4 — Intraspecific body size frequency distribution (IaBSFD) data for insects, indicating whether one or more figures of the data are provided, the outcome of tests for skewness, or normality if former not available, and whether additional data on variation associated with age, sex, time or space are provided. (DOC) [file pone.0016606.s006.doc]

**Supporting Information Table S4.** Intraspecific body size frequency distribution (IaBSFD) data for insects, indicating whether one or more figures of the data are provided, the outcome of tests for skewness, or normality if former not available, and whether additional data on variation associated with age, sex, time or space are provided.

| **Order** | **Species** | **Mass/linear; figure** | **Normality/skew** | **Additional data** | **Source** |
| --- | --- | --- | --- | --- | --- |
| **Isoptera** |  |  |  |  |  |
| Hodotermitidae | *Microhodotermes viator* | Both; yes | Positive skew | - | This study |
| **Orthoptera** |  |  |  |  |  |
| Gryllidae | *Gryllus bimaculatus* | Both; yes | Positive skew | Sex | This study |
| **Hemiptera** |  |  |  |  |  |
| Lygaeidae | *Nysius* sp. | Both; yes | No skew |  | This study |
| Vellidae | *Rhagovelia maculate* | Both; yes | Negative skew | Sex | This study |
| **Lepidoptera** |  |  |  |  |  |
| Satyridae | *Dira clytus* | Both; yes | Positive skew | Sex | This study |
| **Coleoptera** |  |  |  |  |  |
| Apionidae | *Setapion provincial* | Both; yes | No skew | - | This study |
|  | *Setapion quantillum* | Both; yes | No skew |  | This study |
| Cerambycidae | *Phoracantha semipunctata* | Linear; yes | Normal distribution | Lab vs. field | Hanks et al. 2005 |
| Chrysomelidae | Chrysomelid sp. | Both; yes | Positive skew | Sex | This study |
| Coccinellidae | *Coccinella septempunctata* | Linear; yes | Normal distribution; no test | Sex | Evans 2000 |
|  | *Coccinella transversoguttata* | Linear; yes | Normal distribution; no test | Sex | Evans 2000 |
|  | *Henosepilachna vigintioctopunctata* | Both; yes | No skew | Sex | This study |
| Curculionidae | *Ectemnorhinus marioni* | Linear; yes | Normal; no test | Cryptic species | Chown & Smith 1993 |
|  | *Ectemnorhinus similis* | Linear; yes | Normal; no test | Cryptic species | Chown & Smith 1993 |
|  | *Ectemnorhinus viridis* | Linear; yes | Normal distribution; no test | Sex | Chown & Klok 2001 |
|  | *Gonipterus scutellatus* | Both; yes | No skew | Sex | This study |
| Lucanidae | *Prosopocoilus inclinatus* | Mass, but body parts only; yes | Variable skew; K-S tests for normality | Sex | Tatsuta et al. 2004 |
| Scarabaeidae | *Pachnoda sinuata* | Both; yes | No skew | Sex | This study |
| **Diptera** |  |  |  |  |  |
| Culicidae | *Aedes albifasciatus* | Linear; yes | Variable skew; no test | Season | Gleiser et al. 2000 |
|  | *Anopheles aquasalis* | Linear; no | Negative skew | Population | Lounibos 1994 |
|  | *Anopheles albitarsis* | Linear; no | Negative skew | Population | Lounibos 1994 |
|  | *Anopheles nuneztovari* | Linear; no | No skew | Population | Lounibos 1994 |
|  | *Anopheles triannulatus* | Linear; no | No skew |  | Lounibos 1994 |
| Drosophilidae | *Drosophila melanogaster* | Linear | No skew; no test | Lab vs. field | David et al. 1997 |
| Scathophagidae | *Scathophaga stercoraria* | Linear; yes | Normal or negative skew; no test | Sex, mating location | Pitnick et al. 2009 |
| Tephritidae | *Ceratitis capitata* | Both; yes | Positive skew | Sex | This study |
| **Hymenoptera** |  |  |  |  |  |
| Anthophoridae | *Centris pallida* | Linear; yes | Positive skew, no test | Interannual variation | Alcock 1984 |
| Bombicidae | *Bombus hortorum* | Linear; yes | Positive skew, no test |  | Peat et al. 2005 |
|  | *Bombus impatiens* | Linear; no | Variable skew - changes with time | Age | Couvillon et al. 2010 |
|  | *Bombus impatiens* | Linear; yes | Positive skew | Starvation | Couvillon & Dornhaus 2010 |
|  | *Bombus lapidarius* | Linear; yes | Positive skew; no test | Population | Peat et al. 2005 |
|  | *Bombus lucorum* | Linear; yes | Positive skew; no test | Population | Peat et al. 2005 |
|  | *Bombus pascuorum* | Linear; yes | Positive skew; no test | Population | Peat et al. 2005 |
|  | *Bombus pratorum* | Linear; yes | Positive skew; no test |  | Peat et al., 2005 |
|  | *Bombus terrestris* | Linear; yes | No skew; no test | Population | Peat et al. 2005 |
| Formicidae | Formicidae sp. | Both; yes | Positive skew | Sex | This study |
|  | *Acromyrmex echinatior* | Linear; yes | Positive skew; no test | Genetics of polymorphism | Hughes et al. 2003 |
|  | *Atta cephalotes* | Linear; yes | Positive skew; no test | Polyethism | Stradling 1978 |
|  | *Atta cephalotes* | Linear; yes | Positive skew; no test | Age | Wilson 1983 |
|  | *Atta columbica* | Mass; yes | Positive skew; no test | Allometry | Feener et al. 1988 |
|  | *Atta texana* | Linear; yes | Normal to weakly bimodal; no test | Allometry | Wilson 1953 |
|  | *Camponotus abdominalis* | Linear; yes | Bimodal; no test | Allometry | Wilson 1953 |
|  | *Camponotus castaneus* | Linear; yes | Positive skew, weakly bimodal; no test | Allometry | Wilson 1953 |
|  | *Camponotus consobrinus* | Linear; no | Bimodal | Allometry | Fraser et al. 2000 |
|  | *Eciton hamatum* | Mass; yes | Positive skew; no test | Allometry | Feener et al. 1988 |
|  | *Formica exsectoides* | Linear; yes | Normal; no test | Allometry | Wilson 1953 |
|  | *Leptothorax rugatulus* | Linear; yes | Normal to bimodal; no test | Caste | Rüppell & Heinze 1999 |
|  | *Messor pergandei* | Linear; yes | Positive skew; no test | Population | Davidson 1978 |
|  | *Messor pergandei* (as *Veromessor pergandei*) | Linear; yes | Normal, but platykurtic | Foraging efficiency | Rissing & Pollock 1984 |
|  | *Myrmecocystus mexicanus* | Linear; yes | Bimodal; no test | Colony manipulation | Rissing 1984 |
|  | *Oecophylla smaragdina* | Linear; yes | Bimodal; no test | Allometry | Wilson 1953 |
|  | *Paraponera clavata* | Linear; yes | Positive skew | Colony | Breed & Harrison 1988 |
|  | *Pheidole bicarinata* | Linear; yes | Bimodal; no test | Physiological mechanisms | Wheeler & Nijhout 1983 |
|  | *Pheidole rhea* | Linear; yes | Bimodal; no test | Allometry | Wilson 1953 |
|  | *Pheidole subdentata* | Linear; yes | Bimodal | Caste polymorphism | Wheeler 1991 |
|  | *Pogonomyrmex badius* | Linear; yes | Bimodal, leptokurtic; no test | Season, colony size | Tschinkel 1998 |
|  | *Solenopsis invicta* | Linear; no | None to positive skew with age, no test | Age | Wood & Tschinkel 1981 |
|  | *Solenopsis invicta* | Linear; yes | Normal to bimodal (appears skewed); normal scores | Age | Tschinkel 1988 |
|  | *Solenopsis invicta* | Linear; yes | Positive skew; no test | Polymorphism | Wheeler 1991 |
| Pteromalidae | *Trichilogaster acaciaelongifoliae* | Both; yes | No skew | Sex | This study |
|  | *Trichilogaster signiventris* | Both; yes | No skew | Sex | This study |
| Vespidae | *Polistes* sp. | Both; yes | Positive skew |  | This study |

**References:** Alcock 1984 Evolution 38: 220-223; Breed & Harrison 1988 J. Kansas Entomol. Soc. 61: 285-291; Chown & Klok 2001 Polar Biol. 24: 706-712.; Chown & Smith 1993 Oecologia 96: 508-516; Couvilon & Dornhaus 2010 Insect. Soc. 57: 193-197; Couvilon et al. 2010 Ecol. Entomol. 35: 424-435; David et al. 1997 J. Thermal Biol. 22: 441-451; Davidson 1978 Am. Nat. 112: 523-532; Evans 2000 Eur. J. Entomol. 97: 469-474; Feener et al. 1988 Funct. Ecol. 2: 509-520; Fraser et al. 2000 Behav. Ecol. Sociobiol. 47: 188-194; Gleiser et al. 2000 Med. Vet. Entomol. 14: 38-43; Hanks et al. 2005 Entomol. Exp. Appl. 114: 25-34; Hughes et al. 2003 Proc. Natnl Acad. Sci. U.S.A. 100: 9394-9397; Lounibos 1994 Ecol. Entomol. 19: 138-146; Peat et al. 2005 Funct. Ecol. 19:145-151; Pitnick et al. 2009 Proc. R. Soc. B. 276: 3229-3237; Rissing 1984 J. Kansas Entomol. Soc. 57: 347-350; Rissing & Pollock 1984 Behav. Ecol. Sociobiol. 15: 121-126; Rüppell & Heinze 1999 Insect. Soc. 46: 6-17; Stradling 1978 J. Animal Ecol. 47: 173-188; Tatsuta et al. 2004 Biol. J. Linn. Soc. 81: 219-233; Tschinkel 1988 Behav. Ecol. Sociobiol. 22: 103-115; Tschinkel 1998 Insect. Soc. 45: 385-410; Wheeler 1991 Am. Nat. 138: 1218-1238; Wheeler & Nijhout 1983 J. Insect Physiol. 11: 847-854; Wilson 1953 Quart. Rev. Biol. 28: 136-156; Wilson 1983 Behav. Ecol. Sociobiol. 14: 55-60; Wood & Tschinkel 1981 Insect. Soc. 28: 117-128.
